# Association of gross virus-associated cell-surface antigen with liposomes.

**DOI:** 10.1038/bjc.1980.34

**Published:** 1980-02

**Authors:** F. Sakai, D. Gerlier, J. F. Doré

## Abstract

**Images:**


*Br. J. Cancer* (1980) **41**, 227

### ASSOCIATION OF GROSS VIRUS-ASSOCIATED CELL-SURFACE ANTIGEN WITH LIPOSOMES

F. SAKAI, D. GERLIER AND J.-F. DORÉ

*From the Laboratoire d'Immunologie et de Cancérologie Expérimentale, INSERM FRA NO. 24, 69373 Lyon Cedex 2, France*

Received 29 June 1979 Accepted 22 October 1979

**Summary.—Gross Cell-Surface Antigen (GCSAa) was obtained from W/Fu (C58NT)D lymphoma cells by Nonidet P40(NP40) or 3M KCl extraction and further purified by Sephadex G200 filtration. GCSAa was associated with lipids (dipalmitoylphosphatidycholine, cholesterol and dicetylphosphate, in molar ratios of 7:2:1) to form multilamellar liposomes. The amount of protein associated with liposomes was found to be proportional to the protein concentration of the sensitizing cellular extract and to the amount of phospholipids used and, under defined conditions, 22–55% of the protein of the cellular extract could be associated with liposomes. Analysis of disrupted sensitized liposomes showed that the GCSAa-specific activity of the liposome-associated proteins was quite similar to that of the proteins of the sensitizing cellular extract. Ultracentrifugation of disrupted liposomes showed that about 75% of the liposome-associated GCSAa activity was firmly associated with lipids and that little GCSAa was trapped within aqueous compartments between lipidic lamellae. 1·8–8·0% of the liposome-associated GCSAa was expressed at the liposome surface. No striking differences in degree of GCSAa association were found between liposomes sensitized by NP40 or by 3M KCl extracts. Storage experiments at +4°C showed that GCSAa-sensitized liposomes were fairly stable.**

RECENT INTEREST has centred on the use of liposomes as agents for the presentation of antigens to the immune system. Liposomes have been shown to be powerful adjuvants for a variety of antigens (Kinsky & Nicolotti, 1977). This adjuvant effect has been documented with respect to antibody response for proteins such as diphtheria toxin (Allison & Gregoriadis, 1974) and bovine serum albumin (Heath *et al.*, 1976; Van Rooijen & Van Nieuwmegen, 1977). Therefore, incorporation of tumour cell-surface antigens into liposomes by mimicking cell-surface presentation could prove to be of utmost interest, and indeed such a presentation of glycolipid tumour antigens which are readily used for liposome formation has been found to induce tumour rejection (Huet & Ansel, 1977). Preliminary results have shown that incorporation into liposomes of a tumour-associated cell-surface antigen of protein nature could be achieved (Gerlier *et al.*, 1978).

Since Gross virus-associated cell-surface antigen (GCSAa) is immunogenic in W/Fu rats (Geering *et al.*, 1966; Herberman, 1972) we attempted to incorporate into liposomes this membrane protein antigen, which has been extracted from syngeneic (C58NT)D lymphoma cells, in order to study its *in vivo* immunogenicity. The aim of the present work was to investigate the incorporation, expression and distribution of GCSAa in liposomes, and the stability of this association. Biochemical and immunological analysis of sensitized liposomes showed that good yields of incorporation of this antigen into liposomes could be obtained, and that liposomes containing antigenically active GCSAa were stable for several days.

### MATERIAL AND METHODS

*Animals and tumours.*—Inbred W/Fu/RhoIco rats and inbred C57BL/6/RhoIco mice were bred in our colony. Gross-virus-induced (C58NT)D lymphoma (Geering *et al.*) was maintained by weekly passages into syngeneic weanling W/Fu rats. Gross-virus-induced E♂G2 lymphoma was also transplanted weekly into syngeneic C57BL/6 mice (Old *et al.*, 1965).

*Antiserum.*—A pool of syngeneic W/Fu rat antiserum was produced by s.c. immunization with viable (C58NT)D lymphoma cells as previously described (Gerlier *et al.*, 1977*a*). Such an antiserum exclusively detects GCSAa when used in a complement-dependent cytotoxicity test against E♂G2 target cells (Geering *et al.*, 1966; Gerlier *et al.*, 1977*b*; Herberman, 1972).

*Antigen preparation.*—GCSAa was extracted from W/Fu rat (C58NT)D lymphoma cells using the following methods.

(C58NT)D lymphoma cells, previously washed with Dulbecco's phosphate-buffered saline and kept frozen at −70°C, were incubated at a concentration of $5 \times 10^8$ cells/ml in 0·05M phosphate-buffered 0·15M NaCl (pH 7·4) containing 0·5% (v:v) Nonidet P40 (NP40) detergent for 30 min at 4°C.

After centrifugation for 3 h at 48,000 *g* in a Beckman J21B centrifuge, the supernatant was precipitated by 60% ammonium sulphate for 1 h at 4°C, and centrifuged for 15 min at 13,000 *g*. The pellet was dissolved in phosphate buffer, dialyzed for 24 h at 4°C, concentrated 10-fold on a Minicon B15 membrane (Amicon) and centrifuged for 3 h at 48,000 *g*. The supernatant was applied on to a Sephadex G200 column. As previously described (Gerlier *et al.*, 1978) most of the GCSAa activity was eluted from the gel in a fraction containing molecular species of 120,000–60,000 mol. wt, which is in agreement with the finding that GCSAa is borne by glycosylated precursors of virus nucleocapsid proteins of 95,000 and 85,000 mol. wt (Tung *et al.*, 1977; Snyder *et al.*, 1977; Ledbetter & Nowinski, 1977). The GCSAa-containing fractions were pooled and concentrated on a Minicon B15 membrane to reach a concentration of the extract equivalent to $5 \times 10^9$ cells/ml.

Alternatively (C58NT)D lymphoma cells were disrupted by 3M KCl. This was achieved by suspending $10^8$ cells/ml in 0·05M phosphate buffer, 3M KCl, and incubating the mixture for 16 h at 4°C (Reisfeld & Pellegrino, 1971). After dialysis for 24 h at 4°C and centrifugation for 3 h at 48,000 *g*, the extract was treated as above. As controls, NP40 and 3M KCl solubilizations of normal rat lymphoid cells (spleen, thymus and lymph node) were done.

*Assay of GCSAa.*—Gross cell-surface antigen (GCSAa) activity was determined by inhibition of the cytotoxic activity of the anti-(C58NT)D serum on E♂G2 lymphoma target cells, as previously described (Gerlier *et al.*, 1977*a*). Briefly, 50 μl of serum diluted 1:100 (*i.e.*, two dilutions above its 50% titre) were incubated with 50 μl of serial dilutions of soluble antigen for 30 min at 4°C, 20°C or 45°C with or without intermittent stirring; 50 μl of E♂G2 cell suspension in Hepes-buffered Hanks' solution ($4 \cdot 10^6$ cells/ml) was then added for 30 min at 4°C; after a washing with 1·5 ml buffer, the cells were resuspended in 100 μl of an appropriate dilution of selected rabbit complement and further incubated for 30 min at 37°C. Cell viability was measured by trypan-blue dye exclusion. GCSAa activity was expressed as μg protein of the extract able to inhibit 50% of the cytotoxic activity of 50 μl of the antiserum diluted 1:100.

*Preparation of liposomes.*—A homogeneous film of lipids was formed by evaporating chloroform solutions of DL-α-dipalmitoylphosphatidycholine (DPPC, Sigma) cholesterol (Sigma) and dicetylphosphate (DCP, Sigma) in 7:2:1 molar ratios, at 45°C under a $N_2$ stream and further desiccating under vacuum. The lipid film was dispersed by intermittent stirring and heating at 43·5°C for 2 min in 5mM phosphate buffer (pH 7·2) (empty liposomes) or in a cellular extract previously dialyzed against 5mM phosphate buffer and ultracentrifuged at 250,000 *g* for 60 min (sensitized liposomes). The milky suspension was kept at room temperature for 2 h, then diluted with ice-cold 5mM phosphate-buffered saline (PBS) (pH 7·2) and centrifuged at 31,000 *g* for 30 min. Liposomal pellets were resuspended in PBS and washed ×4. Washed liposomes were generally immediately analyzed or, in storage experiments, resuspended in a small volume of PBS and kept at 4°C for one to several days.

*Analysis of liposomes.*—Pelleted liposomes were resuspended in 5mM phosphate buffer (pH 7·2) and allowed to swell at room temperature for 20 min. The suspension (15–33 mg phospholipid/$\mu$l) was kept frozen at $-70$°C until used. To assess the repartition of proteins and GCSAa activity among the liposomal structures, sensitized liposomes were mechanically disrupted by sonication (10–16 intermittent bursts of 30 sec, 20 kHz, 45 W) at 0°C. The resulting suspension was referred as "disrupted liposomes". Disrupted liposomes were further ultracentrifuged (30 min at 160,000 *g* in a Beckman Airfuge) and the supernatant was considered as representing the aqueous-phase constituents.

(a) Biochemical analysis

Several aliquots of suspensions of intact or disrupted liposomes or of the supernatants of the latter were put on a glass-fibre filter. Phospholipids were extracted, washed and assayed according to a modified Bartlett procedure (Portoukalian *et al.*, 1977) and proteins were quantified according to Kruski & Narayan (1972). Proteins of the cellular extract, as well as liposomal proteins, leaked in 5mM PBS were assayed by the Lowry method.

(b) Immunological analysis

The assay of GCSAa activity in disrupted liposomes and their supernatants was performed as described above. As a reference, the original antigenic extract was processed as for incorporation into liposomes (*i.e.*, heated and sonicated) and assayed in parallel.

GCSAa expressed at the liposome surface was determined by adsorption of 100 $\mu$l of the antiserum diluted 1:200 (one dilution above its 50% cytotoxicity titre) with decreasing amounts of freshly washed pelleted intact liposomes, under intermittent stirring at 0°C, 23°C or 45°C. Liposomes were then pelleted again and the residual cytotoxic activity of the serum was determined (Gerlier *et al.*, 1977*a*).

(c) Electron microscopy

Aliquots of liposomes were placed on Formvar-carbon-coated grids, allowed to adhere for 1 min, and then negatively stained with a 2% solution of potassium phosphotungstate for 20 sec. The samples were observed under a Siemens Elmiskop 102 electron microscope.

RESULTS

*Inclusion of proteins in liposomes*

Liposomes were formed with cellular extracts and preliminary experiments showed that, for a given protein concentration of the cellular extract, the amount of liposome-associated protein is directly proportional to the amount of liposomal phospholipid, and to the volume of the liposomal pellet, 90% of which, according to Bangham *et al.* (1967) represents the volume of liposomes. When liposomes were formed in the absence of negatively charged DCP, their volume was 1·9× smaller and they contained 6·7× less protein than those formed in the presence of DCP.

The amount of protein associated with liposomes, as expressed by their protein/phospholipid ratio, was studied, and found to increase linearly when the protein concentration of the cellular extract varied from 3 to 23 mg/ml, independently of the origin or mode of preparation of the extracts (Fig. 1).

From these studies, standard conditions were delineated for liposome formation (40 mg phospholipid and 8–19 mg protein/

FIG. 1.—Association of proteins with liposomes as a function of protein concentration of the sensitizing cellular extracts. Numbers refer to experiments reported in Tables.

FIG. 2.—Electron micrograph of liposomes sensitized by an NP40 extract of (C58NT)D cells. Negative staining with 2% phosphotungstate.

ml of sensitizing cellular extract). Under these conditions, the yield of protein associated to liposomes averaged 40% (22–55%) of the protein of the cellular extract.

*Morphology and constitution of sensitized liposomes*

The liposomes formed under these conditions appeared as a heterogeneous population of bodies, variable in size and shape, which, under the electron microscope, showed a concentric multilamellar structure (Fig. 2). Similar structures were seen for liposomes sensitized either by 3M KCl or NP40 cellular extracts.

When sensitized liposomes were disrupted by 5–8 min of sonication and ultracentrifuged, an average of 25·5% (range 15·4–36·4%) of the liposomal proteins but only an average of 2·5% (range 1·4–3·9%) of the liposomal phospholipids were recovered in the supernatant (Table I). Thus, after sonication, most of the disrupted liposomes sedimented upon ultracentrifugation and appeared as membrane fragments under electron-microscope examination (Tyrell *et al.*, 1976).

TABLE I.—*Relative protein and phospholipid composition of aqueous phases of sensitized liposomes*

| Sensitizing (C58NT) D cell extract | Exp. No. | Proteins of supernatant*/ Total liposome protein (%) | Phospholipid of supernatant*/ Total liposome phospholipid (%) |
|---|---|---|---|
| NP40 | 3 | 36·4 | 2·8 |
| | 4 | 26·8 | 2·3 |
| | 6 | 28·9 | 3·9 |
| | 7 | 15·4 | n.d.† |
| 3M KCl | 11 | 27·0 | 2·4 |
| | 12 | 23·8 | 2·2 |
| | 13 | 18·5 | 1·4 |

* Ultracentrifugation supernatant from disrupted liposomes.

† Not determined.

Ultracentrifugation pellets of these disrupted liposomes sensitized either by an NP40 cellular extract (Exp. 4) or by a 3M KCl cellular extract (Exp. 12) were washed, resuspended in 5mM phosphate buffer (22·7 and 11·3 mg phospholipid/ml respectively) further sonicated for 10 to 30 min and ultracentrifuged. Percentages of protein and phospholipid recovered in the supernatants were now of the same order of magnitude, and reached 21 and

TABLE II.—*Association of GCSAa with liposomes sensitized with NP40 cellular extracts*

| Sensitizing cellular extract | Exp. No. | Absorption temperature (°C) | GCSAa activity* of: Sensitizing cellular extract | Sensitized disrupted liposomes | Supernatant of disrupted liposomes |
|---|---|---|---|---|---|
| (C58NT)D lymphoma cells | 1 | 23 | 13·1 | 26·5 | 15·2 |
| | 2 | 0 | | 60·7 | |
| | | 23 | 19·1 | 45·1 | |
| | | 45 | | 33·2 | |
| | 3 | 23 | 11·2 | | |
| | | 45 | 16·5 | | 10·3 |
| | 4 | 23 | 13·9 | | |
| | | 45 | 16·3 | | 21·2 |
| | 5 | 0 | 17·2 | 106·6 | |
| | | 23 | 19·1 | 39·7 | |
| | | 45 | 21·2 | 28·1 | |
| | | 45† | | 19·9 | |
| | 6 | 0 | 9 | 18·5 | |
| | | 23 | 10·1 | 15·2 | 7·9 |
| | | 45 | 11·4 | 14·5 | 8·3 |
| | | 45† | | 9·2 | |
| | 7 | 23 | 16·0 | 42·2 | 11·7 |
| | | 45 | 16·6 | 38† | 19·7 |
| | 9 | 45 | 10·4 | 22·9† | |
| | 9a§ | 45 | | 31·4† | |
| | 9b§ | 45 | | 29·5† | |
| Normal lymphoid cells | 10 | 23 | (>48·7)‡ | (>61·8)‡ | |
| | | 45† | | (>61·8)‡ | |

* Results are expressed as μg protein absorbing 50% of the initial activity of 50 μl anti-(C58NT)D serum diluted 1:100.
† With stirring.
‡ No measurable GCSAa activity.
§ GCSAa activity assayed after storage at 4°C for 4 days (9a) or 7 days (9b).

30% in Exps 4 and 12 respectively, after 30 min of this additive sonication.

*GCSAa association with liposomes*

Liposomes sensitized by NP40 on 3M KCl (C58NT)D cell extracts were disrupted by sonication and then assayed for GCSAa activity. When they were allowed to react with the reference antiserum at increasing assay temperatures, it was found that GCSAa activity could be best detected at 45°C under stirring (Tables II and III). Under these latter conditions, in most experiments, the specific activity of GCSAa associated with liposomes was slightly lower (Exp. 11 and 13) or equivalent (Exp. 5, 6 and 12) to that of the GCSAa in the sensitizing (C58NT)D cell extract. No major difference was seen between liposomes sensitized either by NP40 (Table II) or 3M KCl (Table III) cellular extracts.

When sensitized liposomes were disrupted and ultracentrifuged, the assay of GCSAa activity in the supernatant was not affected by the assay temperature. The same temperature independence was observed when GCSAa activity was assayed in the sensitizing cellular extract (Tables II and III).

The supernatants from disrupted liposomes sensitized with an NP40 extract, exhibited a GCSAa specific activity somewhat higher than that of the sensitizing extract in 4/7 assays (Table II) whilst the supernatants from disrupted liposomes sensitized by a 3M KCl extract exhibited a GCSAa specific activity lower than that of the sensitizing extract (7/7 assays, Table III).

When empty liposomes or liposomes sensitized by extracts of normal lymphoid cells were similarly disrupted, no GCSAa activity could be detected (Tables II and III, Exps 10 and 15).

TABLE III.—*Association of GCSAa with liposomes sensitized with* 3M *KCl cellular extracts*

| | | | GCSAa activity of | | |
|---|---|---|---|---|---|
| Sensitizing cellular extract | Exp. No. | Absorption temperature (°C) | Sensitizing cellular extract | Sensitized disrupted liposomes | Supernatant of disrupted liposomes |
| (C58NT)D | 11 | 23 | 9·2 | | 19·9 |
| lymphoma cells | | 45* | | 13·4 | |
| | 12 | 0 | 11·8 | 18·9 | 19·0 |
| | | 23 | 11·8 | 17·8 | 17·1 |
| | | 45 | 11·8 | 11·9 | 15·4 |
| | | 45* | | 10·9 | |
| | 13 | 0 | 10·0 | 33·0 | 18·5 |
| | | 23 | 10·9 | 23·3 | 15·1 |
| | | 45 | 9 | 15·5* | 18·5 |
| Normal | 15 | 23 | (>43·5) | (>78) | |
| lymphoid cells | | 45* | | (>78) | |

* With stirring.

TABLE IV.—*GCSAa expression at the surface of liposomes sensitized by NP*40 *cellular extracts*

| | | | GCSAa activity | | |
|---|---|---|---|---|---|
| Sensitizing cellular extract | Exp. No. | Absorption temperature (°C) | Sensitizing cellular extract (A) | Sensitized liposomes (B) | A/B (%) |
| (C58NT)D | 1 | 23 | 13·1 | 210 | 6·2 |
| lymphoma cells | 6 | 0 | 9·0 | 248 | 3·6 |
| | | 23 | 10·1 | 248 | 4·1 |
| | 7 | 23 | 16·0 | 343 | 4·7 |
| | 8 | 23 | 12·5 | 161 | 7·8 |
| | 9 | 0 | 7·3 | 401 | 1·8 |
| | | 23 | 7·3 | 316 | 2·3 |
| | | 45 | 7·3 | 155 | 4·7 |
| Normal lymphoid cells | 10 | 23 | (>61·8) | (>488) | — |

*GCSAa expression at the liposome surface*

Liposomes sensitized by NP40 or 3M KCl (C58NT)D cell extracts were able to absorb the activity of the reference antiserum, whereas this activity was absorbed neither by empty liposomes (data not shown) nor by liposomes sensitized by normal lymphoid cell extracts (Tables IV and V).

No or little difference between absorption at 0°C and 23°C was found (Exps 6 and 9) but at 45°C (*i.e.*, above the transition temperature of DPPC) the absorption was clearly enhanced (Table IV).

The ratio of the amount of proteins of the sensitizing (C58NT)D cell extract to the amount of liposomal proteins able to absorb 50% of the initial activity of the antiserum, is given in Table III. This ratio (1·8–8·0%) gives an estimation of the percentage of GCSAa expressed at the surface of liposomes, since it can be assumed that the specific activities of GCSAa in liposomes and in the initial sensitizing extract are the same. The same amount of GCSAa is available at the surface of liposomes sensitized either by 3M KCl or NP40 extracts.

*Stability of the association of proteins and GCSAa with liposomes*

The leakage of proteins and GCSAa from intact liposomes stored at 4°C in 5 mM PBS was studied in Exps 9 and 14. In Exp. 9, the percentages of leaked proteins on Days 2, 4 and 7 were respectively

TABLE V.—*GCSAa expression at the surface of liposomes sensitized by* 3M *KCl cellular extracts*

| Sensitizing cellular extract | Exp. No. | Absorption temperature (°C) | GCSAa activity: Sensitizing cellular extract (A) | Sensitized liposomes (B) | A/B (%) |
|---|---|---|---|---|---|
| (C58NT)D | 11 | 23 | 10·7 | 203 | 5·3 |
| lymphoma cells | 12 | 23 | 11·8 | 147 | 8·0 |
| | 14 | 23 | 10·9 | 294 | 3·7 |
| Normal lymphoid cells | 15 | 23 | (> 78) | (> 548) | — |

TABLE VI.—*GCSAa expression at the liposome surface after storage at 4°C*

| Exp. No. | Sensitizing (C58NT)D cell extract | Storage time (days) | GCSAa activity*: Sensitizing cellular extract (A) | Sensitized liposomes (B) | A/B (%) |
|---|---|---|---|---|---|
| 9 | NP40 | 0 | 7·3 | 316 | 2·3 |
| | | 2 | — | 337 | 2·2 |
| | | 4 | — | 322 | 2·3 |
| | | 7 | — | 478 | 1·5 |
| 14 | 3M KCl | 0 | 10·9 | 294 | 3·7 |
| | | 1 | — | 294 | 3·7 |
| | | 3 | — | 294 | 3·7 |

* All absorption assays at 23°C.

2·9, 3·7 and 5·0%; in Exp. 14, on Days 1 and 3, they were respectively 4·7 and 5·3%. The GCSAa-specific activity of leaked proteins was the same as that of the sensitizing cellular extract.

GCSAa activity associated with liposomes kept 4 and 7 days at 4°C was found to be 63% and 71% respectively of the freshly assayed GCSAa activity (Table II, Exp. 9).

The expression of GCSAa at the liposome surface remained unaffected upon 3–4 days' storage at 4°C, but showed some decrease after 7 days' storage (Table VI, Exps 9 and 14).

### DISCUSSION

Cell-surface proteins have already been associated with liposomes in a way mimicking their presentation at the cell surface (Curman *et al.*, 1978; Engelhard *et al.*, 1978; Turner & Sanderson, 1978). In view of the potential adjuvant effect of proteins associated with multilamellar liposomes (Allison & Gregoriadis, 1974) we attempted to incorporate a tumour-associated cell-surface antigen, the proteic GCSAa, into multilammelar liposomes formed as described by Gregoriadis *et al.* (1971).

The analysis of liposomes sensitized by (C58NT)D lymphoma-cell extracts tends to document the localization of proteins inside liposomal structures. Since about 25% of the associated proteins but only 2·5% of the phospholipids could be liberated from sensitized liposomes by mechanical disruption, it appears that these proteins are thus trapped in the aqueous compartments between lipidic lamellae, and have virtually no interaction with lipids.

Conversely, several results favour the existence of strong associations between most of the liposome-associated proteins and the lipids: firstly, the analysis of ultracentrifugation pellets of liposomes mechanically disrupted by sonication shows that proteins and lipids are released at the same rate by further sonication; secondly, as measured in Exps 2, 3 and 4, the protein concentration of the sensitizing cellular extract decreases by about 50% after the liposome formation (data not shown) and thirdly, when liposomes were formed in the absence of DCP, the amount of associated proteins was lowered much more than the liposome volume. Hence, it is likely that polar interactions may exist between proteins and lipids within liposomes, as documented by Tyrell *et al.* (1976), which do not exclude hydrophobic interactions, and, in the present experimental conditions (*i.e.*, liposome formation at high concentrations of proteins and lipids) such interactions are highly probable.

(C58NT)D lymphoma-cell extract-sensitized liposomes contain GCSAa activity which appears to be mostly cryptic, since such liposomes expressed at their surface about 5% of liposome-associated GCSAa activity. Most of the liposome-associated GCSAa activity could be detected after sonication disruption of liposomes below the DPPC transition temperature (41°C), which implies liposome structural defects according to Lawaczeck *et al.* (1976) and assay at 45°C.

The recovery of a specific GCSAa activity equivalent to that of the sensitizing cellular extract, indicates no preferential incorporation of either GCSAa-bearing molecules or the unrelated protein molecules in the extract. Thus, the amount of GCSAa activity incorporated into liposomes could be the same as that of the incorporation of the bulk of proteins from the cellular extract (*i.e.* $\sim$40%). Nevertheless, an optimal recovery of GCSAa activity in disrupted liposomes could not always be obtained; this may be due to an absence of reproducibility in the sonication conditions, though some degradation of GCSAa activity during incorporation cannot be excluded.

From studies of supernatants of disrupted liposomes, it can be estimated that about 75% of the liposome-associated GCSAa activity is strongly associated with lipids, since further sonication of disrupted liposomes only releases protein together with lipids, and that only a minor proportion is actually trapped in the liposomal aqueous compartments between lipidic lamellae.

In the present studies, antigenic extracts were obtained both by NP40 and 3M KCl extraction from (C58NT)D cells; no striking difference was observed, either in their ability to be included into liposomes or in their specific GCSAa activity when associated with liposomes. However, the partition of proteins and GCSAa activity between the liposome compartments may differ slightly according to the type of antigenic extract used. A tendency to a concentration of NP40-extracted GCSAa into the aqueous compartments, and to a preferential association of 3M KCl GCSAa with lipidic lamellae, was seen in most experiments (Tables II & III). This could be related to differences between the properties of GCSAa molecules obtained by the two extraction procedures: firstly, some GCSAa molecules obtained by NP40 extraction could exist in micellar form (Helenius & Simons, 1975); secondly, GCSAa molecules obtained by 3M KCl extraction could have been exposed to some proteolytic degradation, which modified the size and polar properties of the native GCSAa molecules (Mann, 1972).

Several concomitant data show that GCSAa association with liposomes is fairly stable. As a matter of fact, storage experiments showed low leakage of GCSAa into the suspending medium, a slight decrease of GCSAa activity at the liposome surface, and a good recovery of the initial liposome-associated GCSAa activity within several days.

Thus, under the experimental conditions described here, sensitization of liposomes either by NP40 or by 3M KCl extracts of (C58NT)D lymphoma cells allows much incorporation into liposomal structure of antigenically active, mostly cryptic and stable GCSAa. Most of the GCSAa activity is strongly associated with lipids, and this might mimic a membrane presentation of the antigen.

The authors wish to thank J. Portoukalian and F. Audubert for helpful discussions and advice, and H. Cabrillat, M. Groleas, M. Resvoy (Unité de Morphologie Cellulaire et Tissulaire, Centre Léon Bérard) and M. F. Jacquier (INSERM U.51) for electron-microscopy studies.

This work was supported by INSERM (CRL 78.4.186.2) and partly supported by DGRST (Grant No. 75.7.1369).

### REFERENCES

ALLISON, A. C. & GREGORIADIS, G. (1974) Liposomes as immunological adjuvants. *Nature*, **252**, 252.

BANGHAM, A. D., DE GIER, J. & GREVILLE, G. D. (1967) Osmotic properties and water permeability of phospholipid liquid crystals. *Chem. Phys. Lipids*, **1**, 225.

CURMAN, B., ÖSTBERG, L. & PETERSON, P. A. (1978) Incorporation of murine MHC antigens into liposomes and their effect in the secondary mixed lymphocyte reaction. *Nature*, **272**, 545.

ENGELHARD, V. H., GUILD, B. C., HELENIUS, A., TERHORST, C. & STROMINGER, J. L. (1978) Reconstitution of purified detergent-soluble HLA-A and HLA-B antigens into phospholipid vesicles. *Proc. Natl Acad. Sci., U.S.A.*, **75**, 3230.

GEERING, G., OLD, L. J. & BOYSE, E. A. (1966) Antigens of leukaemias induced by naturally occurring murine leukaemia virus: Their relation to the antigen of Gross virus and other murine leukaemia viruses. *J. Exp. Med.*, **124**, 753.

GERLIER, D., GUIBOUT, C. & DORÉ, J. F. (1977*a*) Highly cytotoxic antisera obtained in W/Fu rats against a syngeneic Gross virus induced lymphoma. *Eur. J. Cancer*, **13**, 855.

GERLIER, D., GUILLEMAIN, B., DORÉ, J. F. & DUPLAN, J. F. (1977*b*) Expression d'un antigène associé au virus de Gross à la surface de cellules murines productrices d'un oncornavirus des radioleucémies de la souris C57BL/6. *C. R. Acad. Sci. [D] Paris*, **284**, 2431.

GERLIER, D., SAKAI, F. & DORÉ, J. F. (1978) Inclusion d'un antigène de surface cellulaire associé au virus de Gross dans des liposomes. *C. R. Acad. Sci. [D] Paris*, **286**, 439.

GREGORIADIS, G., LEATHWOOD, P. D. & RYMAN, B. E. (1971) Enzyme entrapment in liposomes. *FEBS. Lett.*, **14**, 95.

HEATH, T. D., EDWARDS, D. C. & RYMAN, B. E. (1976) The adjuvant properties of liposomes. *Biochem. Soc. Trans.*, **4**, 129.

HELENIUS, A. & SIMONS, K. (1975) Solubilization of membranes by detergents. *Biochim. Biophys. Acta*, **415**, 29.

HERBERMAN, R. B. (1972) Serological analysis of cell surface antigens of tumours induced by murine leukaemia virus. *J. Natl Cancer Inst.*, **48**, 265.

HUET, C. & ANSEL, S. (1977) SV40 tumour rejection induced by vesicular stomatitis virus bearing SV40 tumour-specific transplantation antigen (SV40-TSTA). II. Association of SV40-TSTA activity with liposomes containing VSV glycolipids. *Int. J. Cancer*, **20**, 61.

KINSKY, S. C. & NICOLOTTI, R. A. (1977) Immunological properties of model membranes. *Ann. Rev. Biochem.*, **46**, 49.

KRUSKI, A. W. & NARAYAN, K. A. (1972) A simplified procedure for protein determination of turbid lipoprotein samples. *Anal. Biochem.*, **47**, 299.

LAWACZECK, R., KAINOSHO, M. & CHAN, S. I. (1976) The formation and annealing of structural defects in lipid bilayer vesicles. *Biochim. Biophys. Acta*, **443**, 313.

LEDBETTER, J. & NOWINSKI, R. C. (1977) Identification of the Gross cell surface antigen associated with murine leukaemia virus infected cells. *J. Virol.*, **23**, 315.

MANN, D. L. (1972) The effect of enzyme inhibitors on the solubilization of HL-A antigens with 3M KCl. *Transplantation*, **14**, 398.

OLD, L. J., BOYSE, E. A. & STOCKERT, E. (1965) The G (Gross) leukaemia antigen. *Cancer Res.*, **25**, 813.

PORTOUKALIAN, J., BUGAND, M., ZWINGELSTEIN, G. & PRECAUSTA, P. (1977) Comparison of the lipid composition of rabies virus propagated in NIL 2 cells maintained in monolayer versus spinner culture. *Biochim. Biophys. Acta*, **489**, 106.

REISFELD, R. A. & PELLEGRINO, M. A. (1971) Salt extraction of soluble HL-A antigens. *Science*, **172**, 1134.

SNYDER, H. W., STOCKERT, E. & FLEISSNER, E. (1977) Characterization of molecular species carrying Gross cell surface antigen. *J. Virol.*, **23**, 302.

TUNG, J. S., PINTER, A. & FLEISSNER, E. (1977) Two species of type C viral core polyprotein on AKR mouse leukemia cells. *J. Virol.*, **23**, 430.

TURNER, M. J. & SANDERSON, A. R. (1978) The preparation of liposomes bearing human (HLA) transplantation antigens. *Biochem. J.*, **171**, 505.

TYRELL, D. A., HEATH, T. D., COLLEY, C. M. & RYMAN, B. E. (1976) New aspects of liposomes. *Biochim. Biophys. Acta*, **457**, 259.

VAN ROOIJEN, N. & VAN NIEUWMEGEN, R. (1977) Liposomes in immunology: the immune response against antigen-containing liposomes. *Immunol. Comm.*, **6**, 489.


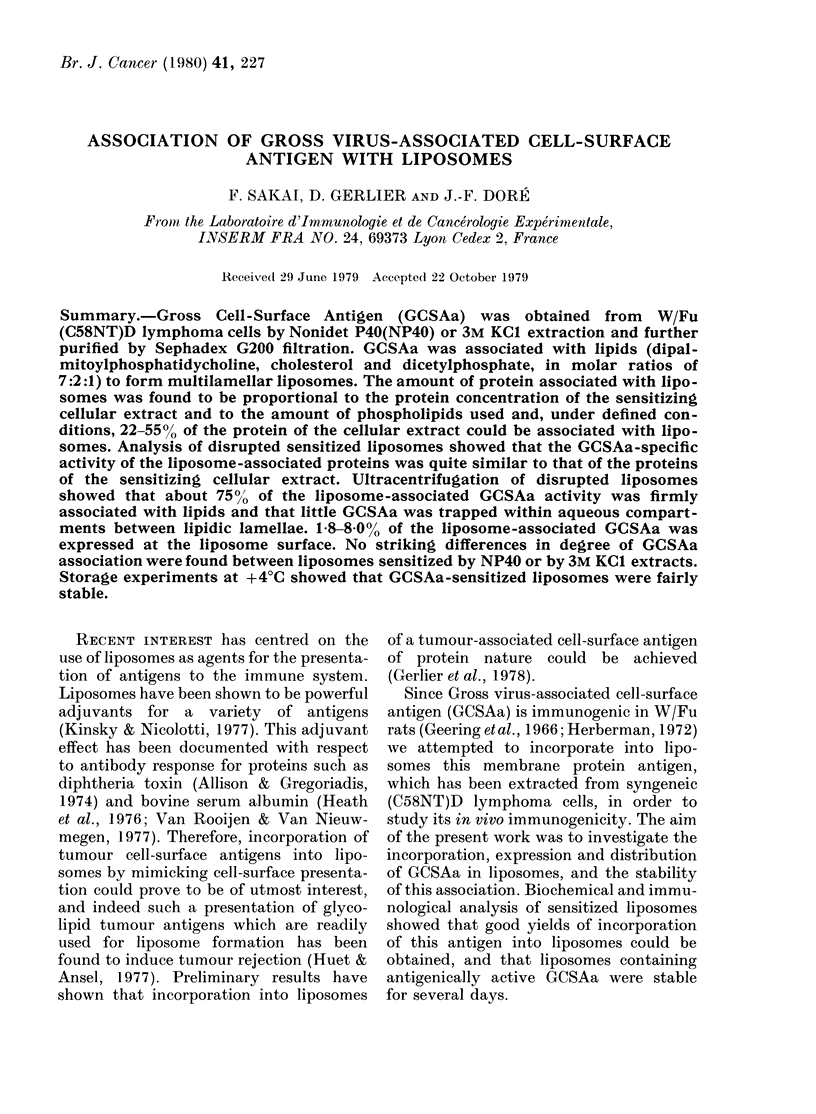

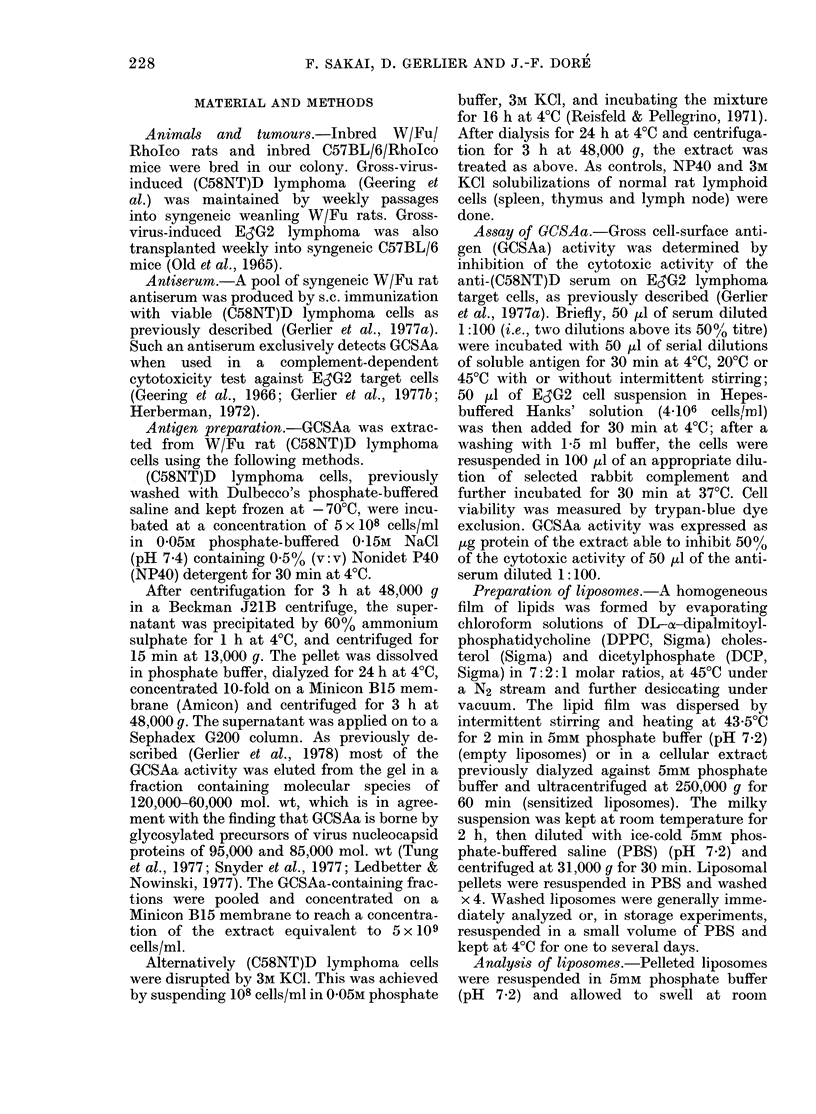

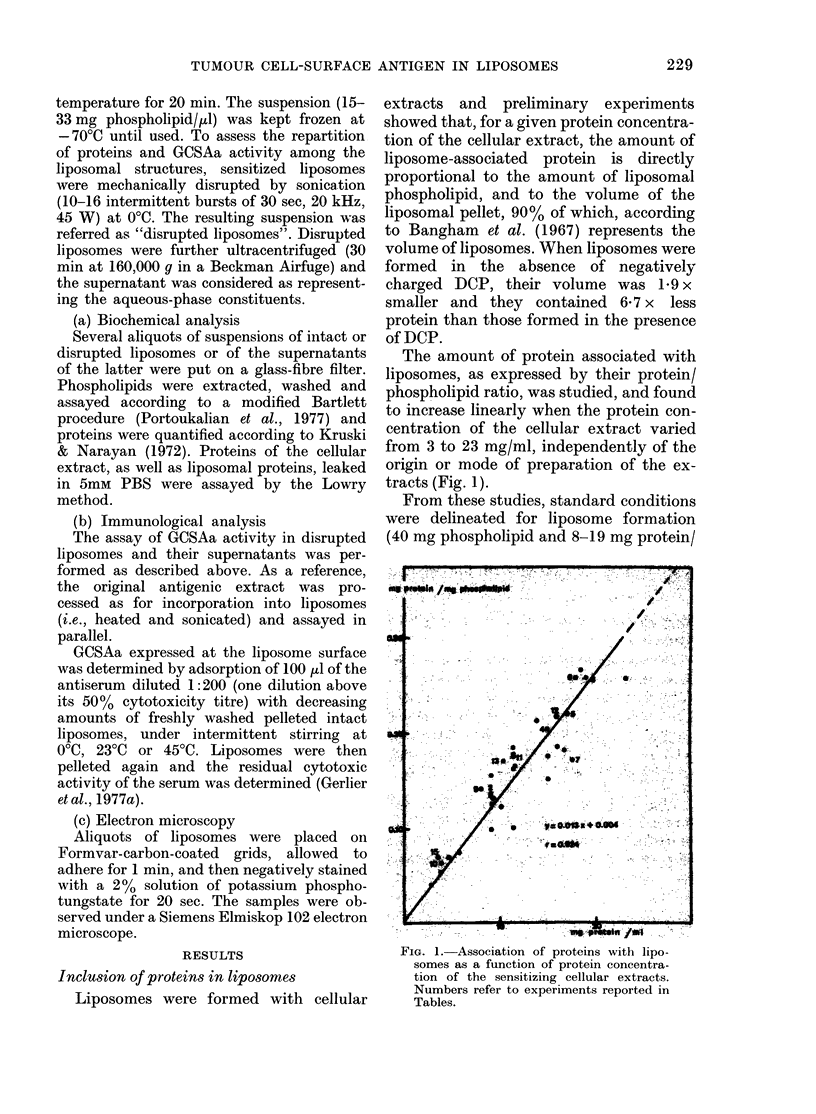

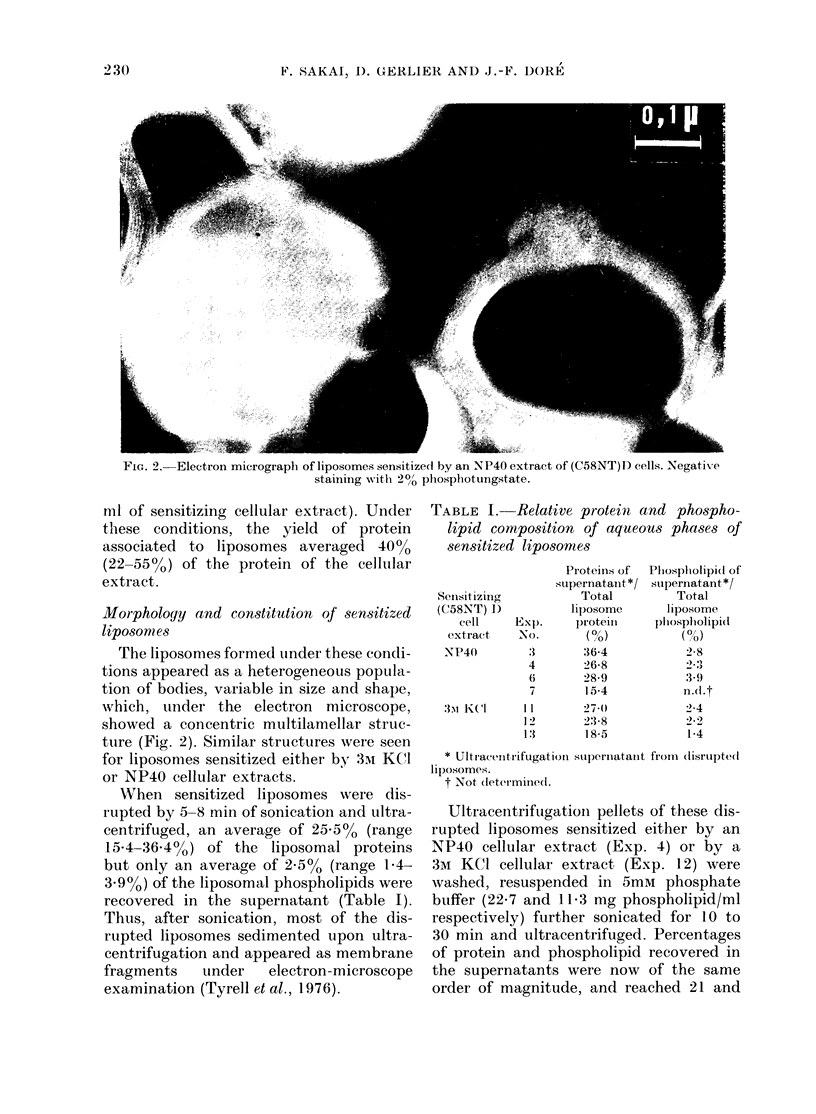

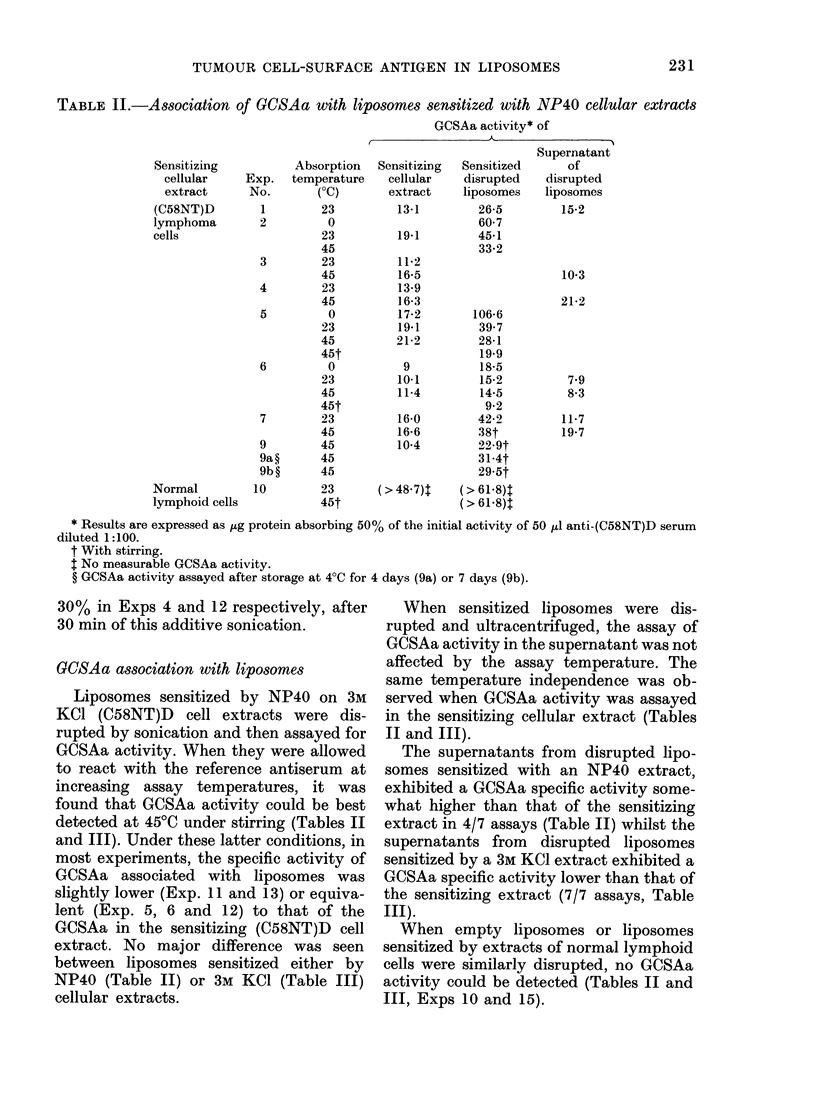

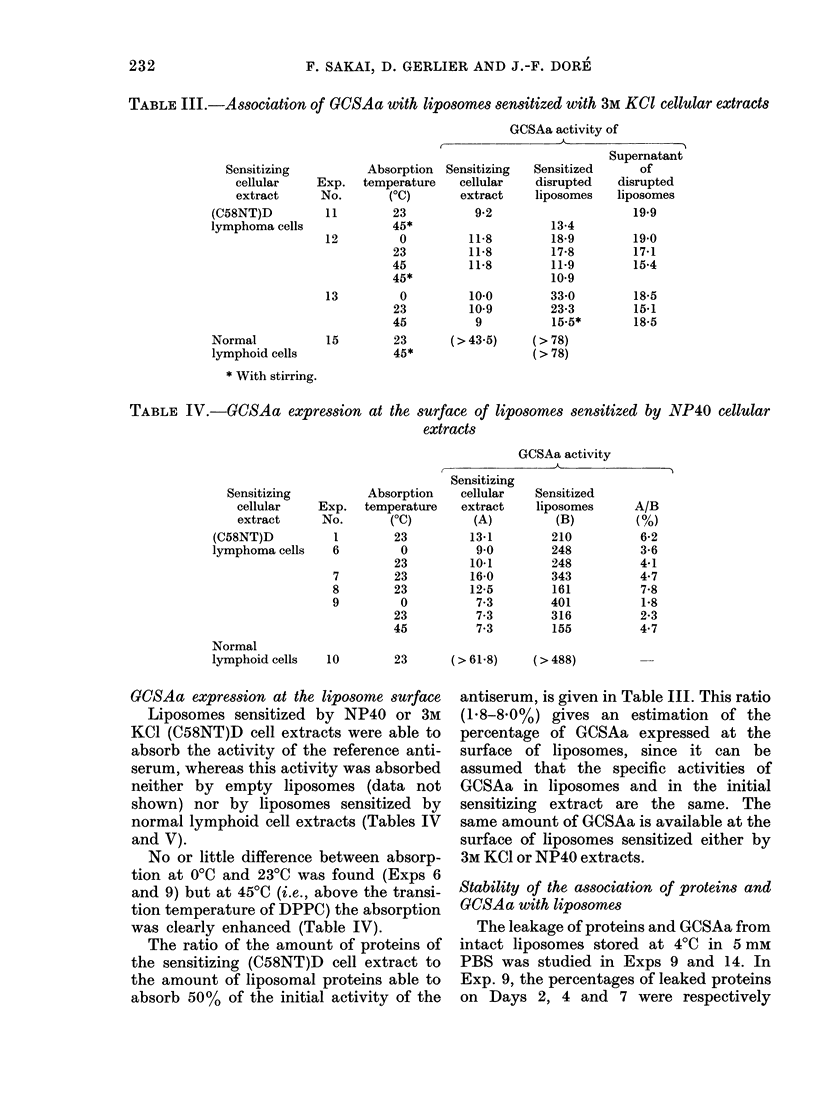

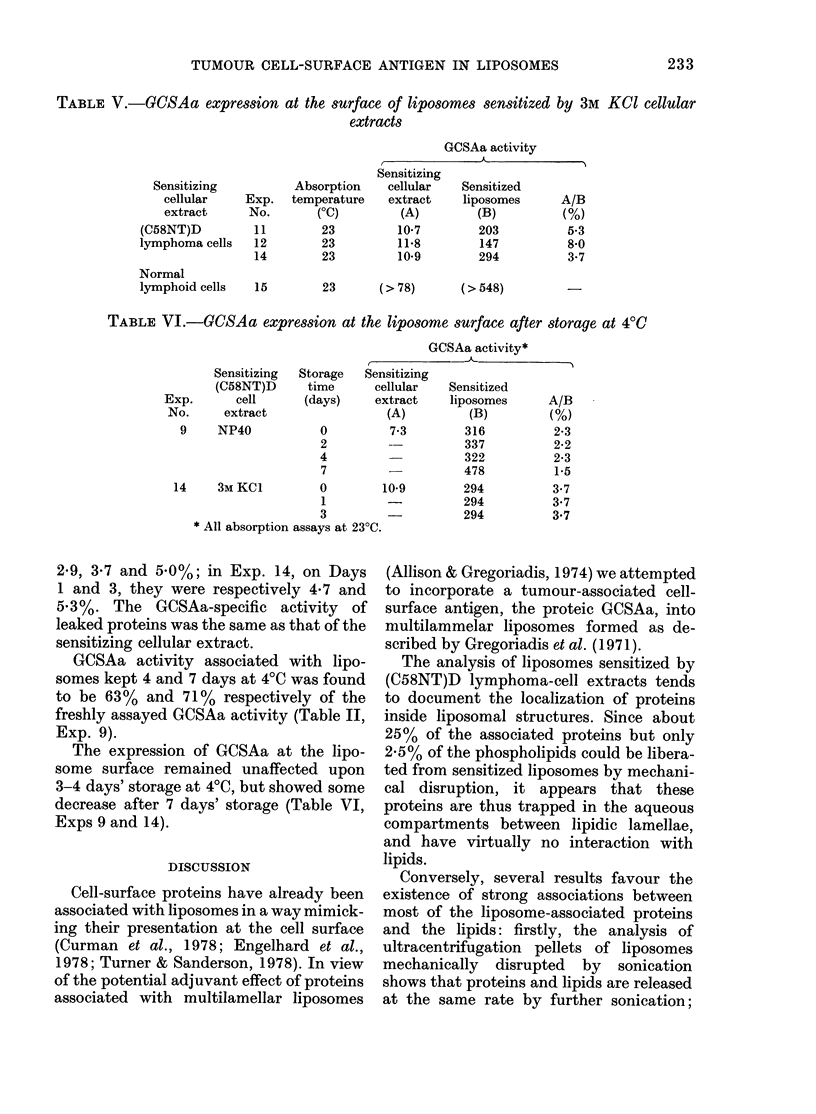

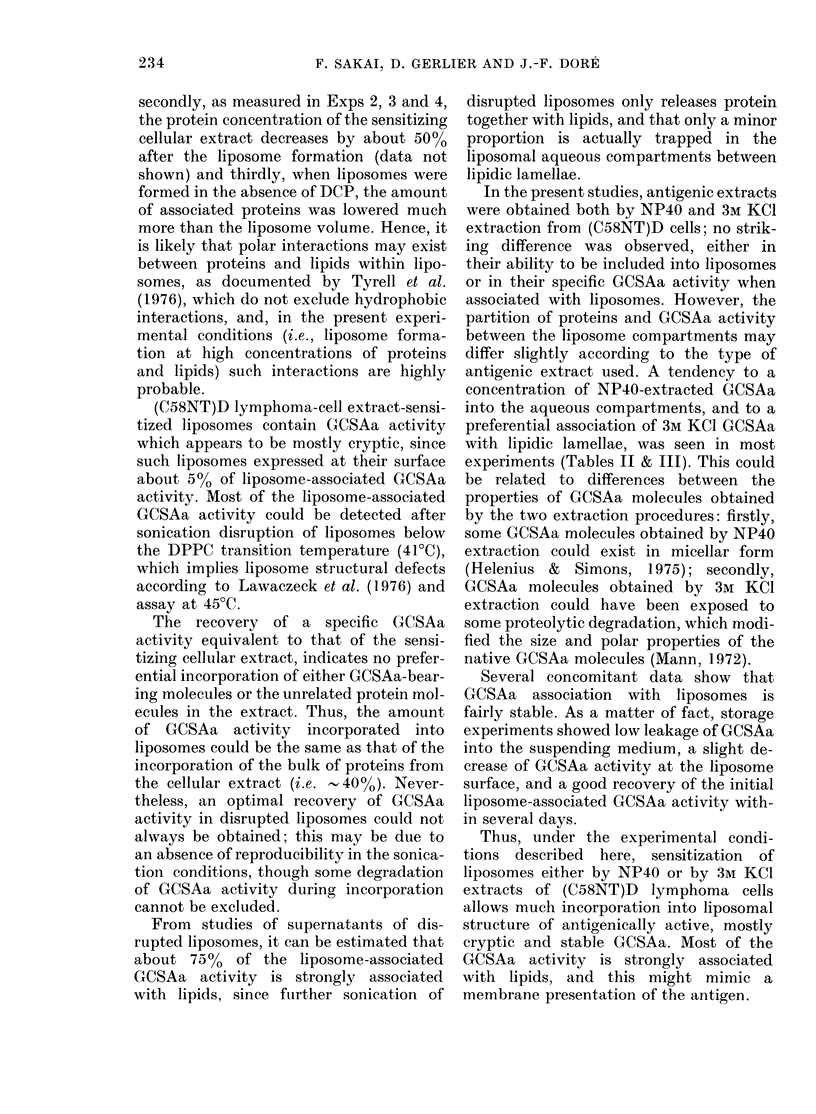

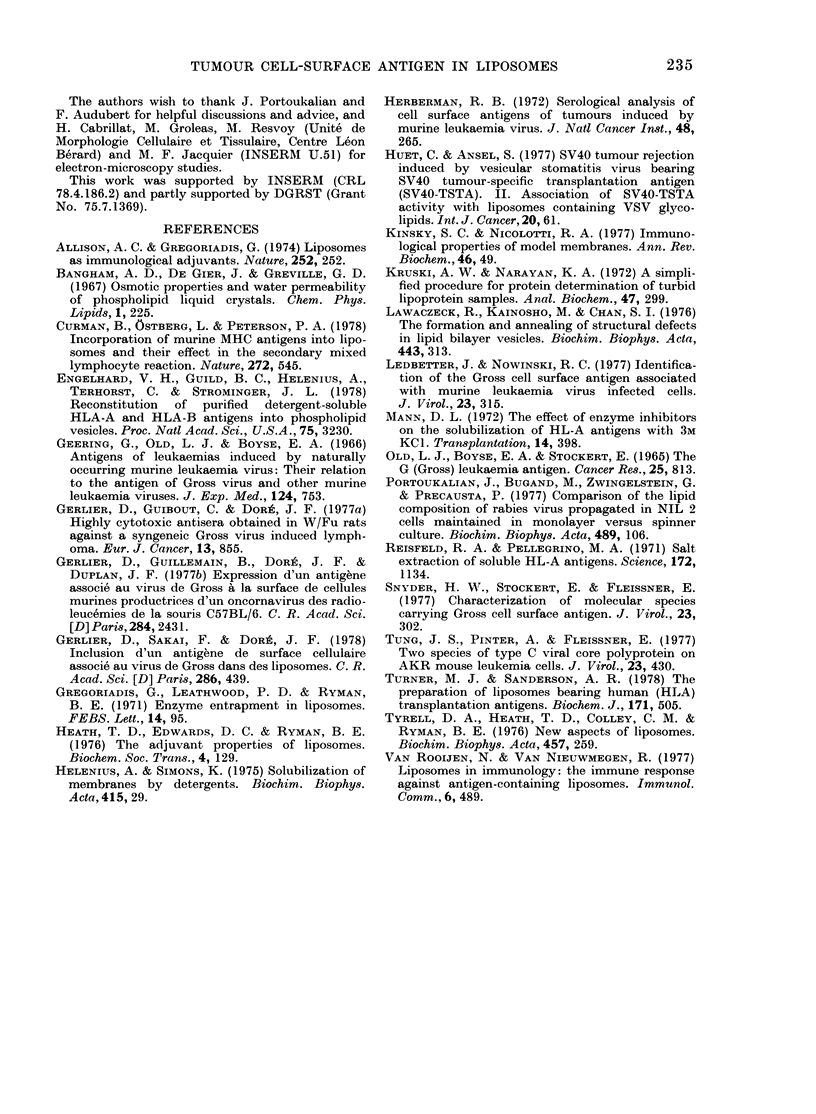


## References

[OCR_01119] Allison A. G., Gregoriadis G. (1974). Liposomes as immunological adjuvants.. Nature.

[OCR_01129] Curman B., Ostberg L., Peterson P. A. (1978). Incorporation of murine MHC antigens into liposomes and their effect in the secondary mixed lymphocyte reaction.. Nature.

[OCR_01135] Engelhard V. H., Guild B. C., Helenius A., Terhorst C., Strominger J. L. (1978). Reconstitution of purified detergent-soluble HLA-A and HLA-B antigens into phospholipid vesicles.. Proc Natl Acad Sci U S A.

[OCR_01142] Geering G., Old L. J., Boyse E. A. (1966). Antigens of leukemias induced by naturally occurring murine leukemia virus: their relation to the antigens of gross virus and other murine leukemia viruses.. J Exp Med.

[OCR_01149] Gerlier D., Guibout C., Dore J. F. (1977). Highly cytotoxic antisera obtained in W/Fu rats against a syngeneic Gross virus induced lymphoma.. Eur J Cancer.

[OCR_01155] Gerlier D., Guillemain B., Doré J. F., Duplan J. F. (1977). Expression d'un antigène associé au virus de gross à la surface de cellules murines productrices d'un oncornavirus des radioleucémies de la souris C57BL/6.. C R Acad Sci Hebd Seances Acad Sci D.

[OCR_01163] Gerlier D., Sakai F., Doré J. F. (1978). Inclusion d'un antigène de surface cellulaire associé au virus de Gross dans des liposomes.. C R Acad Sci Hebd Seances Acad Sci D.

[OCR_01169] Gregoriadis G., Leathwood P. D., Ryman B. E. (1971). Enzyme entrapment in liposomes.. FEBS Lett.

[OCR_01174] Heath T. D., Edwards D. C., Ryman B. E. (1976). The adjuvant properties of liposomes.. Biochem Soc Trans.

[OCR_01179] Helenius A., Simons K. (1975). Solubilization of membranes by detergents.. Biochim Biophys Acta.

[OCR_01184] Herberman R. B. (1972). Serological analysis of cell surface antigens of tumors induced by murine leukemia virus.. J Natl Cancer Inst.

[OCR_01190] Huet C., Ansel S. (1977). SV40 tumor rejection induced by vesicular stomatitis virus bearing SV40 tumor-specific transplantation antigen (SV40-TSTA). II. Association of SV40-TSTA activity with liposomes containing VSV glycolipids.. Int J Cancer.

[OCR_01198] Kinsky S. C., Nicolotti R. A. (1977). Immunological properties of model membranes.. Annu Rev Biochem.

[OCR_01203] Kruski A. W., Narayan K. A. (1972). A simplified procedure for protein determination of turbid lipoprotein samples.. Anal Biochem.

[OCR_01208] Lawaczeck R., Kainosho M., Chan S. I. (1976). The formation and annealing of structural defects in lipid bilayer vesicles.. Biochim Biophys Acta.

[OCR_01214] Ledbetter J., Nowinski R. C. (1977). Identification of the Gross cell surface antigen associated with murine leukemia virus-infected cells.. J Virol.

[OCR_01220] Mann D. L. (1972). The effect of enzyme inhibitors on the solubilization of HL-A antigens with 3 M KCl.. Transplantation.

[OCR_01225] Old L. J., Boyse E. A., Stockert E. (1965). The G (Gross) leukemia antigen.. Cancer Res.

[OCR_01228] Portoukalian J., Bugand M., Zwingelstein G., Precausta P. (1977). Comparison of the lipid composition of rabies virus propagated in Nil 2 cells maintained in monolayer versus spinner culture.. Biochim Biophys Acta.

[OCR_01235] Reisfeld R. A., Pellegrino M. A., Kahan B. D. (1971). Salt extraction of soluble HL-A antigens.. Science.

[OCR_01240] Snyder H. W., Stockert E., Fleissner E. (1977). Characterization of molecular species carrying gross cell surface antigen.. J Virol.

[OCR_01246] Tung J. S., Pinter A., Fleissner E. (1977). Two species of type C viral core polyprotein on AKR mouse leukemia cells.. J Virol.

[OCR_01251] Turner M. J., Sanderson A. R. (1978). The preparation of liposomes bearing human (HLA) transplantation antigens.. Biochem J.

[OCR_01256] Tyrrell D. A., Heath T. D., Colley C. M., Ryman B. E. (1976). New aspects of liposomes.. Biochim Biophys Acta.

[OCR_01261] van Rooijen N., van Nieuwmegen R. (1977). Liposomes in immunology: the immune response against antigen-containing liposomes.. Immunol Commun.

